# Influence Mechanism of the Affordances of Chronic Disease Management Apps on Continuance Intention: Questionnaire Study

**DOI:** 10.2196/21831

**Published:** 2021-05-13

**Authors:** Yongmei Liu, Fei Jiang, Peiyang Lin

**Affiliations:** 1 Business School of Central South University Changsha City China

**Keywords:** health empowerment, perceived affordances, uses and gratifications, S-O-R framework, continuance intention, chronic disease management app

## Abstract

**Background:**

Mobile health apps are becoming increasingly popular, and they provide opportunities for effective health management. Existing chronic disease management (CDM) apps cannot meet users’ practical and urgent needs, and user adhesion is poor. Few studies, however, have investigated the factors that influence the continuance intention of CDM app users.

**Objective:**

Starting from the affordances of CDM apps, this study aimed to analyze how such apps can influence continuance intention through the role of health empowerment.

**Methods:**

Adopting a stimulus-organism-response framework, an antecedent model was established for continuance intention from the perspective of perceived affordances, uses and gratifications theory, and health empowerment. Perceived affordances were used as the “stimulus,” users’ gratifications and health empowerment were used as the “organism,” and continuance intention was used as the “response.” Data were collected online through a well-known questionnaire survey platform in China, and 323 valid questionnaires were obtained. The theoretical model was tested using structural equation modeling.

**Results:**

Perceived connection affordances were found to have significant positive effects on social interactivity gratification (*t*_717_=6.201, *P*<.001) and informativeness gratification (*t*_717_=5.068, *P*<.001). Perceived utilitarian affordances had significant positive effects on informativeness gratification (*t*_717_=7.029, *P*<.001), technology gratification (*t*_717_=8.404, *P*<.001), and function gratification (*t*_717_=9.812, *P*<.001). Perceived hedonic affordances had significant positive effects on function gratification (*t*_717_=5.305, *P*<.001) and enjoyment gratification (*t*_717_=13.768, *P*<.001). Five gratifications (*t*_717_=2.767, *P*=.005; *t*_717_=4.632, *P*<.001; *t*_717_=7.608, *P*<.001; *t*_717_=2.496, *P*=.012; *t*_717_=5.088, *P*<.001) had significant positive effects on health empowerment. Social interactivity gratification, informativeness gratification, and function gratification had significant positive effects on continuance intention. Technology gratification and enjoyment gratification did not have a significant effect on continuance intention. Health empowerment had a significant positive effect on continuance intention. Health empowerment and gratifications play mediating roles in the influence of affordances on continuance intention.

**Conclusions:**

Health empowerment and gratifications of users’ needs are effective ways to promote continuance intention. The gratifications of users’ needs can realize health empowerment and then inspire continuance intention. Affordances are key antecedents that affect gratifications of users’ needs, health empowerment, and continuance intention.

## Introduction

### Background

Chronic diseases are among the main threats to people’s health; they have complex causes and are difficult to cure. Self-management of health is an effective way to treat and alleviate chronic diseases. Treating chronic diseases through self-management requires individuals to have sufficient health knowledge and access to medical resources. However, the capacity of Chinese community health service institutions for chronic disease management (CDM) is weak, offline professional medical resources are difficult to obtain, and individuals lack professional guidance and know little about chronic disease treatment. With the development of internet technology and mobile phones, people are increasingly using mobile phones to manage their health. Mobile health apps are becoming increasingly popular, and they provide opportunities for effective health management [1]. However, related research has shown that existing CDM apps cannot meet users’ practical and urgent needs, and user adhesion is poor [2]. A review by Triantafyllidis et al [3] found that mobile health interventions show mixed evidence in promoting the efficiency of CDM. The continuous use of CDM apps is a key means for people to manage their diseases and for enterprises to remain competitive. Most previous studies of CDM apps have focused on the design, development, or effectiveness of the apps [4,5]. Few, however, have investigated the factors that influence the continuous use intention of app users. As such, there is a need for further research on the factors that influence the continuance intention of CDM app users.

To achieve the goal of restoring health status through self-management, it is necessary to emphasize the role of health empowerment in self-management [6]. Since health empowerment emphasizes the individual’s sense of control over his or her own health and the improvement of the individual’s health knowledge and capabilities [7], it has a positive effect on health management [8]. Health empowerment can stimulate the individual’s internal potential and health awareness; it is thus an effective strategy for helping individuals to produce healthy behaviors and maximize their level of health [9,10]. Such factors are key to continuous individual behavior. Therefore, it is feasible to explore users’ continuance intention from the perspective of health empowerment. However, existing research on whether and how CDM apps can achieve health empowerment has not provided sufficient answers. Thus far, studies have found that the outcomes of health empowerment can include reinventing the self, stimulating internal strength, promoting self-management, improving quality of life, expanding social support, and promoting healthy behavior [10]. It is not yet known, however, whether health empowerment can promote continuance intention.

Meeting users’ needs is an effective way to realize health empowerment and promote continuance intention [11,12]. Can CDM apps meet the need of users to achieve health empowerment and thus affect continuance intention? In recent years, researchers have increasingly adopted the uses and gratifications theory (UGT) to study continuance intention [13-15]. UGT provides theoretical guidance for us to investigate what needs information technology users have while also providing a theoretical lens for exploring how to promote users’ health empowerment. Previous studies using UGT have tended to start from the gratification of users’ needs while ignoring how those needs are satisfied. Affordance refers to an app’s potential to support certain behaviors, emotions, and cognitions, and it can be perceived by users with relevant needs [16]. When users feel that their needs are supported by technology products, their own needs are met. Therefore, this study used the theoretical perspective of affordances (technical features) to explain how a CDM app can meet users’ different needs. It is also feasible to study the empowerment of technology products by considering their technical features [17]. Further, the gratification of user needs can explain the psychological mechanism through which technical products empower users.

In summary, starting from the affordances of CDM apps, this study aimed to analyze how such apps can influence users’ continuance intention through the role of health empowerment. This study addresses the following 2 questions: First, how do CDM apps meet users’ needs? Second, how do users’ need gratifications and health empowerment affect users’ continuance intention?

### Theoretical Foundation

The stimulus-organism-response (S-O-R) framework was used as the basic framework for the model in this study. Mehrabian and Russell [18] noted that an individual in a specific environment will be stimulated by various factors that will trigger internal changes in the individual, which will lead to a corresponding response by the individual. In the context of this study, we combined affordance, UGT, and health empowerment, using affordances as stimulus variables, user need gratifications and health-empowerment awareness as organism variables, and continuance intention as the response variable.

Affordances are the technical features of CDM apps (environmental stimulus), which provide environmental factors for us to examine how CDM apps affect user need gratification, health empowerment, and continuance intention. Perceived affordance, as an environmental stimulus attribute, is a novel perspective for studying individual behavior [19,20]. At the same time, affordance also provides research ideas for examining how user needs are met. We summarized the comprehensive affordances of CDM apps through 3 kinds of affordances (ie, utilitarian, connective, hedonic) according to Scheepers and Middleton [21].

Menon [22] argued that health empowerment is the individual’s perception of control over his or her health and health care; it reflects the internalization of health ideals and goals on personal and social levels. Londoño and Schulz [8] applied psychological empowerment to the field of health care to obtain a measure of mental health empowerment comprising 4 dimensions: self-determination, meaning, impact, and competence.

UGT mainly focuses on identifying users’ psychological needs when selecting media, and it explains users’ subsequent psychological and behavioral changes according to the degree of gratification [23]. Previous research has used UGT to explore users’ continuance intentions mainly in terms of 4 types of gratification: utilitarian, social, enjoyment, and technology gratification [12]. An important factor in CDM app use is whether the app has suitable functions to support users’ health management (function gratification). Therefore, this study added the unique gratification of “self-management function gratification” as an aspect of utilitarian gratification. The explanation of all constructs of UGT is shown in Table 1.

**Table 1:** The constructs of uses and gratifications theory (UGT).

Gratifications	Construct	Definition in this research
Social	Social interactivity gratification	The degree to which the CDM^a^ app assists a user to establish and maintain contact with other users. For example, through the app, a user can get to know other users (doctors or peers) and interact with emotions and information.
Utilitarian	Informativeness gratification	The degree to which using the CDM app helps manage information or deliver meaning. For example, through the various multimedia modes of the app, one can obtain health knowledge or exchange authentic experiences.
Utilitarian	Function gratification	The extent to which users perceive the CDM app to help them manage diseases and solve problems. For example, CDM apps can guide user management, help develop personalized plans, and acquire knowledge.
Enjoyment	Enjoyment gratification	The degree to which users believe that the CDM app brings relaxation and relief. For example, users can access health resources and other users through the app, thereby improving self-efficacy and alleviating depression.
Technology	Technology gratification	The degree to which the CDM apps enables users to perform health management in a convenient and easy way. For example, the friendly interface and ubiquity of the app allow users to conveniently manage chronic diseases.

^a^CDM: chronic disease management.

### Research Model and Hypotheses

**Figure 1:**
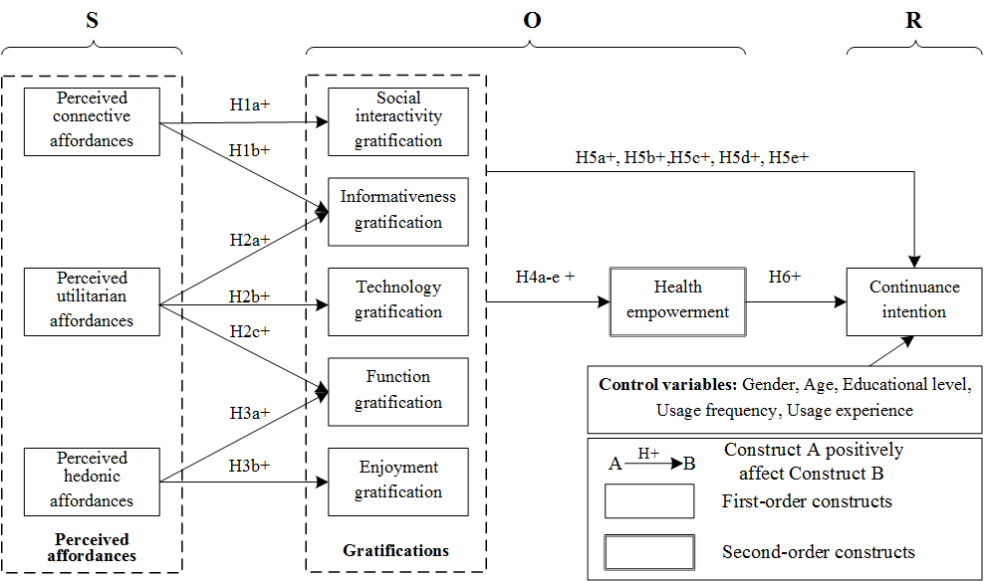
Research model.

Figure 1 shows the research model. When users perceive the different affordances of CDM apps, they will have different degrees of gratification according to how well their needs are supported. Based on different degrees of gratification, users will further make judgments about their health empowerment and willingness to continue using the CDM app. The hypotheses of this study are H1-H6, and detailed information about the hypotheses is shown in Multimedia Appendix 1.

## Methods

### Measurements

This study’s theoretical model has 10 key constructs, and all measurements refer to previously verified scales. The research model contains 3 types of perceived affordances and 5 UGT-related constructs. They are the user's evaluation of the information technology characteristics of CDM apps and the perception of gratifications after use. Their measurements come from scales that have been verified in the field of information systems. The measurement of 3 kinds of perceived affordances referred to research by Tang and Zhang [24] on social e-commerce. Perceived connective affordances refer to how a user perceives the CDM app allows them to interact with other users, and the measurement contains 3 items (eg, “The CDM app has some features that support me to communicate with others”). Perceived utilitarian affordances refers to the action possibility that a user perceives the CDM app helps them improve health management, and the measurement contains 4 items (eg, “The CDM app has some features that support me to collect information related to health management”). Perceived hedonic affordances refers to a user’s perception that the CDM app can make them feel relaxed and relieve anxiety, and the measurement contains 3 items (eg, “The CDM app has some features that that support my relaxation spirit”).

Combining users’ practical needs with UGT, we measured 5 user-need gratifications: social interactivity, informativeness, technology, function, and enjoyment. For the measurement instruments of social interactivity, informativeness, and enjoyment gratification, we referred to research by Kim et al [14] on mobile social network sites [14]. Social interactivity gratification contains a total of 4 items (eg, “The CDM app is satisfying me in helping me maintain social relationships with other users.”). Informativeness gratification contains 4 items (eg, “I am satisfied with using the CDM app to search for various health information.”). Enjoyment gratification contains 4 items (eg, “I am in a good mood when using the CDM app to conduct health management–related activities.”). We followed the process by Gan and Li [12] to measure technology gratification. The measurement by Gan and Li appears to be related to a study about a type of social media app and contains 3 items (eg, “A CDM app is the simplest and most cost-effective way to manage health.”). Moreover, we followed the process by Glasgow et al [25] to measure function gratification, and their measurement appears to be related to a study about chronic illness care. The scale includes 3 items (eg, “The CDM app is satisfying me in helping me customize the treatment plans.”).

We adopted the measurement instruments of Londoño and Schulz [8] to measure health empowerment. The measurement by Londoño and Schulz appears to be related to a study on asthma and showed good reliability and validity. With health empowerment as a second-order reflexive construct in the research model, each first-order factor has 3 items: meaning (eg, “Using the CDM app to meet my health needs is of unique significance to me.”), competence (eg, “I have mastered some of the necessary skills to meet my health needs by using the CDM app.”), self-determination (eg, “I have great autonomy in how to manage health in the process of using the CDM app,”), impact (eg, “My impact on how to manage my own health is great in the process of using the CDM app.”).

Finally, a scale with 3 items was used to measure continuance intention, which referred to the measurement instruments used by Hooi and Cho [26]. Multimedia Appendix 2 lists all the scales used in this study.

Since this study used a Chinese CDM app, the original English questionnaire was translated into Chinese by professional researchers with consideration of the specifics of the app to ensure equivalence in the translation [12]. After the preliminary preparation of the questionnaire, several researchers were invited to review it. Based on their feedback, items were reworded to improve readability and clarity. A 5-point Likert scale was used for all measurements (“disagree very much” to “agree very much”). We also measured relevant demographic information and variables that might affect the dependent variables, including gender, age, education, income, usage experience, and use frequency [27].

### Data Collection

Data were collected online through a well-known questionnaire survey platform in China [28]. This is a professional online survey platform with more than 40 million members and more than 2.6 million sample members. It covers all areas of society and ensures the authenticity of the sample; it has been used and confirmed by a large number of scholars in different fields [23,29]. At the same time, the platform has a sample database of CDM app users, and it issues questionnaires for such users. To further identify suitable responders, we set up some screening questions at the beginning of the questionnaire (eg, “Have you used a related CDM app [Micro Sugar, Hypertension Housekeeper, Diabetes Control, etc.]” “Have you communicated with other users of the CDM app?” “Have you obtained health knowledge through the CDM app?”). Only participants who pass all the screening questions can continue to fill in the questionnaire; otherwise, the ability to answer questions will be terminated. Data collection lasted 1 week, during which a total of 382 samples were collected. Among them, 27 cases were unfinished, accounting for 7.1% of the total (27/382). To ensure the reliability of the questionnaire, we eliminated 19 responses (19/382, 5.0%) for which the questionnaires took less than 5 minutes to complete and 13 responses (13/382, 3.4%) for which the answers consistently showed the same or similar values (which appeared as unlikely responses). Finally, 323 valid questionnaires were obtained.

Cohen power tables were used to test whether the sample size was sufficient to detect the effects of interest [30]. This method is recommended for the power analysis of studies using partial least squares (PLS) [31]. In this study, the largest number of independent variables of the construct (health empowerment and continuance intention) was 5. According to Cohen power tables, assuming a medium eﬀect size (*f*^2^=0.15), statistical power of 0.80, and significance level of .05, the smallest sample size was 91. Therefore, a sample size of 323 was sufficient in our study.

### Data Analysis

A PLS structural equation model was used for data analysis, employing SmartPLS 3.0. PLS is particularly suitable for dealing with multistage models [32]. The measurement model was evaluated through confirmatory factor analysis (CFA); then, the structural model was tested by bootstrapping [33].

## Results

### General Statistical Description

Among the 323 valid questionnaires, the proportions of men and women were 45.0% (145/323) and 55.0% (178/323), respectively. Among the participants, the 31-40-year age group was the largest, accounting for 43.3% (140/323); 68.1% (267/323) of the participants had 1-3 years of experience using a chronic disease app; and 88.9% (287/323) of the participants used it less than 3 times a day. Regarding the level of education, 78.3% (253/323) of the participants had a bachelor's degree. Table 2 shows the detailed demographic information.

**Table 2:** Demographic characteristics (n=323).

Characteristics			Results, n (%)
**Gender**			
	Male	145 (45.0)	
	Female	178 (55.0)	
**Age (years)**			
	18-30	136 (42.1)	
	31-40	140 (43.3)	
	41-50	30 (9.3)	
	51-60	17 (5.3)	
**App use experience (years)**			
	0-1	47 (14.6)	
	1-2	118 (36.5)	
	2-3	102 (31.6)	
	3-4	39 (12.1)	
	4-5	13 (4.0)	
	>5	4 (1.2)	
**App use frequency (times/day)**			
	0-3	287 (88.9)	
	4-6	28 (8.7)	
	≥7	8 (2.5)	
**Educational level**			
	High school or below	9 (2.8)	
	Junior college	37 (11.5)	
	Undergraduate	253 (78.3)	
	Masters or above	24 (7.4)	
**Monthly income (RMB)**			
	<3000	28 (8.7)	
	3000-4999	44 (13.6)	
	5000-7999	92 (28.5)	
	8000-9999	74 (22.9)	
	10,000-14,999	69 (21.4)	
	≥15,000	16 (5.0)	

### Measurement Model

First, we tested the reliability and validity of the constructs in the model through CFA. The factor loadings of all first-order constructs were greater than the recommended value of 0.7 [34]. Health empowerment was a second-order reflexive construct in which the dimension of self-determination was less than the recommended value of 0.7, thus excluding the dimension of self-determination [35]. The minimum values for composite reliability and Cronbach α were 0.827 and 0.715, respectively, which are greater than the recommended value of 0.7, thus ensuring the reliability of the construct measurement [36]. In addition, the average variance extracted (AVE) was higher than the critical value of 0.5 [36]. In summary, these indicators confirm the convergent validity of the measurement model. Multimedia Appendix 3 provides the test results of convergence validity and reliability.

Second, we tested discriminant validity by comparing the square root of the AVE of the constructs with the correlation coefficient between constructs. If the former is greater than the latter, discriminant validity can be proven [37]. The correlations between the constructs were all less than 0.7, and the values on the diagonal (the square root of AVE) were significantly greater than the correlation coefficients between constructs. Therefore, discriminant validity was verified. For details, see Multimedia Appendix 4.

Finally, according to the correlation matrix between constructs, the correlation coefficient between technology gratification and health empowerment was the maximum value (0.608), which is less than the recommended value of 0.8, and the maximum value of all variance inflation factors was 1.793, well below the critical value of 10 [38]. Thus, multicollinearity was not a serious problem in this study. The Harman single-factor test was performed using SPSS version 22 (IBM Corp, Armonk, NY) to analyze method bias. The analysis was performed without rotation, and no factor was specified. If the number of factors extracted is more than one and the variance contribution rate of the first factor does not exceed 40%, it is generally considered that common-method bias is not serious [39]. In this study, the obtained first-factor variance contribution rate was 24.23% (less than 40%); thus, common-method bias was not a serious problem.

### Structural Model

**Figure 2:**
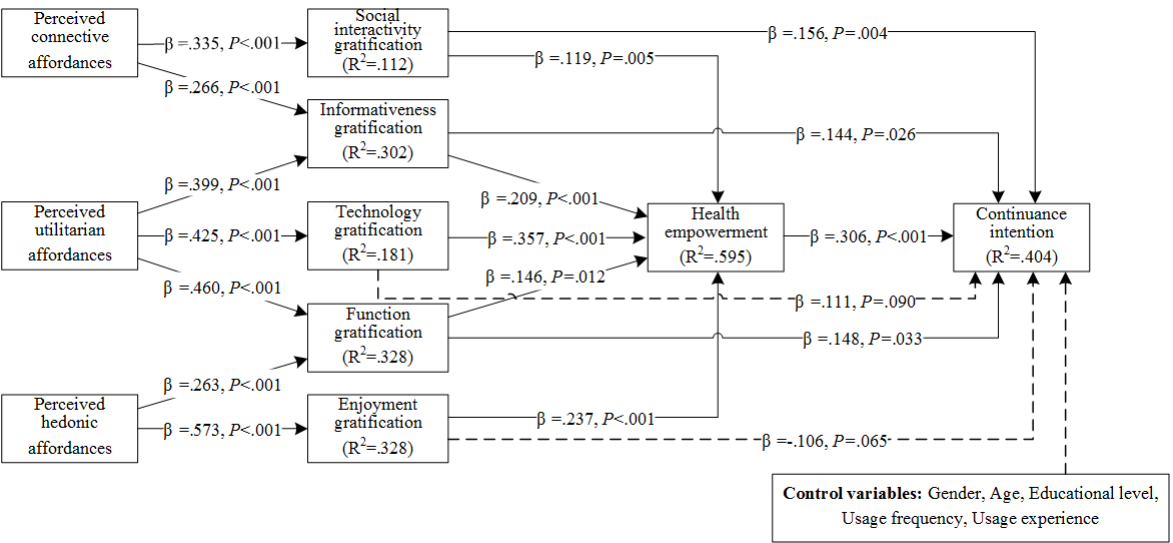
Analysis results of the structural model.

Next, we tested the hypotheses. Figure 2 shows the results. Perceived connection affordances were found to have significant positive effects on social interactivity gratification and informativeness gratification. Thus, H1a and H1b are supported. Perceived utilitarian affordances had significant positive effects on informativeness gratification, technology gratification, and function gratification; thus, H2a, H2b, and H2c are supported. Perceived hedonic affordances had significant positive effects on function gratification and enjoyment gratification. Therefore, H3a and H3b are supported. The gratification of the user’s 5 needs had a significant positive effect on health empowerment. Thus, H4a, H4b, H4c, H4d, and H4e are supported. Social interactivity, informativeness, and function gratification had significant positive effects on continuance intention. Thus, H5a, H5b, and H5d are all supported. Technology gratification and enjoyment gratification did not have a significant effect on continuance intention. Therefore, H5c and H5e are not supported. Health empowerment had a significant positive effect on users’ continuance intention. H6 is therefore supported. Finally, all control variables did not have significant effects on the dependent variable. Overall, the model explained 59.5% of the variation in health empowerment and 40.4% of the variation in continuance intention and thus had good explanatory power.

### Mediating Effect Analysis of Health Empowerment

The bootstrap method was used for the analysis of mediating effects. Compared to traditional methods, the advantage of this method is that it can directly test the indirect effects of independent variables on the dependent variables, and it does not require the mediating effects to follow a normal distribution [40,41]. The sample size was selected as 5000, and the selected confidence interval method was the bias-corrected and accelerated bootstrap. Table 3 summarizes the mediating analysis results obtained using SmartPLS 3.2.8. The effect of perceived connection affordances on continuance intention worked through 4 paths. Therefore, there is evidence that health empowerment played a mediating role in the influence of perceived connection affordances, social interactivity gratification, and informativeness gratification on continuance intention. Perceived utilitarian affordances affected continuance intention through 5 paths. Thus, there is evidence that health empowerment played a mediating role in the influence of perceived utilitarian affordances, informativeness gratification, technology gratification, and function gratification on continuance intention. Perceived hedonic affordances affected continuance intention through 3 paths. Thus, there is evidence that health empowerment mediated the influence of perceived hedonic affordances, function gratification, and enjoyment gratification on continuance intention.

**Table 3:** Bootstrapping results.

Indirect effect path	95% confidence interval	Path of the corresponding direct effect	95% confidence interval	Type of mediation
PCA^a^ - SIG^b^ - CI^c^	0.016 to 0.097	PCA - CI	0.069 to 0.175	Partial
PCA -SIG - HE^d^ - CI	0.003 to 0.027	PCA - CI	0.069 to 0.175	Partial
PCA - IG^e^ - CI	0.006 to 0.075	PCA - CI	0.069 to 0.175	Partial
PCA - IG - HE - CI	0.007 to 0.034	PCA - CI	0.069 to 0.175	Partial
PUA^f^ - IG - CI	0.005 to 0.118	PCA - CI	0.190 to 0.338	Partial
PUA - IG – HE - CI	0.011 to 0.048	PCA - CII	0.190 to 0.338	Partial
PUA - TG^g^ - HE -CI	0.022 to 0.081	PCA - CI	0.190 to 0.338	Partial
PUA - FG^h^ - CI	0.006 to 0.138	PCA - CI	0.190 to 0.338	Partial
PUA - FG - HE - CI	0.004 to 0.049	PCA - CI	0.190 to 0.338	Partial
PHA^i^ - FG - CI	0.005 to 0.088	PCA - CI	–0.041 to 0.110	full
PHA - FG - HE - CI	0.003 to 0.030	PCA - CI	–0.041 to 0.110	full
PHA - EG^j^ - HE - CI	0.018 to 0.074	PCA - CI	–0.041 to 0.110	full

^a^PCA: perceived connection affordances.

^b^SIG: social interactivity gratification.

^c^CI: continuance intention.

^d^HE: health empowerment.

^e^IG: informativeness gratification.

^f^PUA: perceived utilitarian affordances.

^g^TG: technology gratification.

^h^FG: function gratification.

^i^PHA: perceived hedonic affordances.

^j^EG: enjoyment gratification.

## Discussion

### Principal Findings

This study obtained the following important findings: First, we found that CDM apps can achieve health empowerment by gratifying users’ needs through affordances. Gratification is key to health empowerment. Among the affordances of CDM apps, perceived connective, utilitarian, and hedonic affordances help gratify needs related to the connection (social interactivity, informativeness), utilitarian experience (informativeness, self-management, technology), and hedonic experience (enjoyment, function), respectively. Health empowerment gives users a sense of a self-determined environment for generating healthy behaviors. Therefore, it is important for the user’s health that the user understand how the CDM app promotes his or her health empowerment. This finding is the same as those in the studies by Nelson et al [17] and Audrain-Pontevia and Menvielle [42], once again proving that the application of information technology in the health field can effectively promote health empowerment.

Second, health empowerment was found to be a key factor influencing users’ continuance intention, and it mediated the influences of affordance and gratification on continuance intention. This result suggests that enhancing the user’s sense of empowerment is crucial to the lifespan of CDM apps. Promoting the user’s sense of health empowerment is a feasible way to stimulate continuance intention. Different from previous research on health empowerment, which focused on the rights of patients [8-10], this finding shows that health empowerment can also promote the continued use of health-related applications. Promoting health empowerment is a win-win for patients and app providers.

To suit our research scenario, we added “the gratification of self-management functions.” We found that gratification from the self-management function was a major factor influencing users’ health empowerment and continuance intention. Therefore, CDM apps should provide health management functions corresponding to users’ needs to help users manage their own health, thereby improving user satisfaction with the functional design. We also found that technology gratification and enjoyment gratification did not have a significant effect on continuance intention; thus, H5c and H5e were not supported. The possible reason for H5c being rejected is that technology gratification is more concerned with convenience and ease of use; such factors affect the initial adoption of the app but have little effect on continuance intention. Regarding H5e, this study was conducted in the context of CDM; thus, the demand for enjoyment might not be so large relative to other user needs. In addition, when we used bootstrapping to test the mediating effect, we found that its effect on continuance intention was fully mediated by health empowerment.

### Implications for Research

This study’s theoretical contributions are as follows: First, by combining affordance, UGT, and health empowerment, an antecedent model for the intention to continue using CDM apps was established. This provides novel research ideas and a research framework for investigating information system use continuance, supplementing existing research on this topic. This study considered the role of health empowerment as a contextual factor. Unlike previous studies that usually explore the effect of perceived usefulness and satisfaction on continued use intention [43,44], this study focused on CDM apps’ ability to empower users and health empowerment’s effect on continuance intention. This provides a new perspective for exploring the continued use of health applications.

Second, this study provides empirical guidance for studying how to promote health empowerment. It also enriches the literature on factors influencing health empowerment. Considering the important role of health empowerment in stimulating individual health behaviors, this study found that satisfying the different needs of users has a positive effect on users’ health empowerment. Although past research has indicated that health empowerment can be achieved by satisfying individual needs [11,45], this has never been tested empirically in the context of CDM app usage. Future research may consider starting from the perspective of “affordance-gratification-health empowerment” to explore more empowerment methods.

Third, this study expands the application of UGT in information systems literature by considering the role of a new type of gratification and also deepens our understanding of the antecedents of continuance intention. UGT has usually been used to explain why people choose a particular media without involving a CDM app [12,46]. This study found that the gratification of self-management functions was an important factor influencing the continuous use of CDM apps. Further prior research using UGT was based on the gratification of various user needs and ignored the antecedents of gratifications [13-15]. This study revealed how a CDM app can promote the gratification of user needs from the perspective of affordance. Therefore, this study expands UGT research and deepens our understanding of it.

### Limitations and Future Research

This study has some limitations. First, the samples involved Chinese CDM app users. The conclusions should be carefully considered in other scenarios. Comparative cross-cultural and cross-platform studies could be considered in the future. Second, this study considered 3 kinds of affordances for CDM apps based on previous studies. There may be other types of affordances that could be considered; future research could develop more affordances. In addition to the 4 kinds of gratifications scholars have used in the past, this study uniquely added “gratification of self-management needs.” Users may have other types of needs, which should be considered in future research. Third, the data in this study are cross-sectional data, and there is a lack of analysis of influencing factors based on the time span. Longitudinal research can be considered in future studies. Finally, although we have specified the target of the questionnaire and set up screening items, some false respondents may be included in the final sample. Future studies should consider research methods that have more control over the respondents (eg, experimental designs).

### Conclusion

Under the overall framework of S-O-R, this study examined the influence mechanism of CDM app users’ continuance intention based on the perspective of perceived affordance, UGT, and health empowerment. The results indicated that users’ perceptions of an app’s affordances can promote the gratification of needs, and the gratification of key needs (ie, social interactivity, informativeness, technology, and function gratification) can stimulate users’ continuance intention. At the same time, the gratification of users’ needs can promote users’ cognitions of health empowerment, thus stimulating continuance intention. Health empowerment was found to play a mediating role in the influence of gratification on continuance intention. From a practical perspective, app service providers should design apps from the perspective of social interaction (eg, providing social networks), utilitarian functions (eg, health self-management), and hedonic functions (eg, enhancing the user’s interest). By meeting users’ various needs, app developers can improve the user’s ability to control his or her own health, thus achieving the purpose of extending the life of the app.

## Appendix

Multimedia Appendix 1 The detailed information of the hypotheses.

Multimedia Appendix 2 Measurement instruments.

Multimedia Appendix 3 The results of convergence validity and reliability.

Multimedia Appendix 4 Discriminant validity.

## Abbreviations

AVE: average variance extracted
CDM: chronic disease management
CFA: confirmatory factor analysis
PLS: partial least squares
S-O-R: stimulus-organism-response
UGT: uses and gratifications theory

## References

[1] Ernsting C, Dombrowski SU, Oedekoven M, O Sullivan JL, Kanzler M, Kuhlmey A, Gellert P (2017). Using Smartphones and Health Apps to Change and Manage Health Behaviors: A Population-Based Survey. J Med Internet Res.

[2] Brzan PP, Rotman E, Pajnkihar M, Klanjsek P (2016). Mobile Applications for Control and Self Management of Diabetes: A Systematic Review. J Med Syst.

[3] Triantafyllidis A, Kondylakis H, Votis K, Tzovaras D, Maglaveras N, Rahimi K (2019). Features, outcomes, and challenges in mobile health interventions for patients living with chronic diseases: A review of systematic reviews. Int J Med Inform.

[4] van der Weegen S, Verwey R, Spreeuwenberg M, Tange H, van der Weijden T, de Witte L (2013). The development of a mobile monitoring and feedback tool to stimulate physical activity of people with a chronic disease in primary care: a user-centered design. JMIR Mhealth Uhealth.

[5] Cafazzo JA, Casselman M, Hamming N, Katzman DK, Palmert MR (2012). Design of an mHealth app for the self-management of adolescent type 1 diabetes: a pilot study. J Med Internet Res.

[6] Ramsay Wan C, Vo L, Barnes CS (2012). Conceptualizations of patient empowerment among individuals seeking treatment for diabetes mellitus in an urban, public-sector clinic. Patient Educ Couns.

[7] Cerezo PG, Juvé-Udina Maria-Eulália, Delgado-Hito P (2016). Concepts and measures of patient empowerment: a comprehensive review. Rev Esc Enferm USP.

[8] Londoño Ana Maria Moreno, Schulz PJ (2015). Influences of health literacy, judgment skills, and empowerment on asthma self-management practices. Patient Educ Couns.

[9] Aujoulat I, Marcolongo R, Bonadiman L, Deccache A (2008). Reconsidering patient empowerment in chronic illness: a critique of models of self-efficacy and bodily control. Soc Sci Med.

[10] Aujoulat I, d'Hoore W, Deccache A (2007). Patient empowerment in theory and practice: polysemy or cacophony?. Patient Educ Couns.

[11] Anderson RM, Funnell MM, Butler PM, Arnold MS, Fitzgerald JT, Feste CC (1995). Patient empowerment. Results of a randomized controlled trial. Diabetes Care.

[12] Gan C, Li H (2018). Understanding the effects of gratifications on the continuance intention to use WeChat in China: A perspective on uses and gratifications. Computers in Human Behavior.

[13] Lee HE, Cho J (2017). What Motivates Users to Continue Using Diet and Fitness Apps? Application of the Uses and Gratifications Approach. Health Commun.

[14] Kim MJ, Lee C, Contractor NS (2019). Seniors' usage of mobile social network sites: Applying theories of innovation diffusion and uses and gratifications. Computers in Human Behavior.

[15] Hsu MH, Chang CM, Lin HC, Lin YW (2015). Determinants of continued use of social media: the perspectives of uses and gratifications theory and perceived interactivity. Information Research.

[16] Norman DA (1988). The Psychology of Everyday Things.

[17] Nelson EC, Verhagen T, Noordzij ML (2016). Health empowerment through activity trackers: An empirical smart wristband study. Computers in Human Behavior.

[18] Mehrabian A, Russell JA (1974). An approach to environmental psychology.

[19] Seet B, Goh T (2012). Exploring the affordance and acceptance of an e-reader device as a collaborative learning system. The Electronic Library.

[20] Zhao Y, Liu J, Tang J, Zhu Q (2013). Conceptualizing perceived affordances in social media interaction design. AP.

[21] Scheepers R, Middleton C (2013). Personal ICT Ensembles and Ubiquitous Information Systems Environments: Key Issues and Research Implications. CAIS.

[22] Menon ST (2002). Toward a model of psychological health empowerment: implications for health care in multicultural communities. Nurse Educ Today.

[23] Huang J, Zhou L (2018). Timing of web personalization in mobile shopping: A perspective from Uses and Gratifications Theory. Computers in Human Behavior.

[24] Tang J, Zhang P (2020). The impact of atmospheric cues on consumers’ approach and avoidance behavioral intentions in social commerce websites. Computers in Human Behavior.

[25] Glasgow RE, Wagner EH, Schaefer J, Mahoney LD, Reid RJ, Greene SM (2005). Development and validation of the Patient Assessment of Chronic Illness Care (PACIC). Med Care.

[26] Hooi R, Cho H (2017). Virtual world continuance intention. Telematics and Informatics.

[27] Coursaris CK, Yun Y, Sung J (2010). Twitter Users vs. Quitters: A Uses and Gratifications and Diffusion of Innovations Approach in Understanding the Role of Mobility in Microblogging.

[28] wjx.cn. Changsha Ranxing Information Technology Co., Ltd.

[29] Lien CH, Cao Y (2014). Examining WeChat users’ motivations, trust, attitudes, and positive word-of-mouth: Evidence from China. Computers in Human Behavior.

[30] Cohen J (1992). A power primer. Psychol Bull.

[31] Benitez J, Henseler J, Castillo A, Schuberth F (2020). How to perform and report an impactful analysis using partial least squares: Guidelines for confirmatory and explanatory IS research. Information & Management.

[32] Fornell C, Bookstein FL (2018). Two Structural Equation Models: LISREL and PLS Applied to Consumer Exit-Voice Theory. Journal of Marketing Research.

[33] Anderson JC, Gerbing DW (1988). Structural equation modeling in practice: A review and recommended two-step approach. Psychological Bulletin.

[34] Carmines EG, Zeller RA (1979). Reliability and validity assessment.

[35] Shin JI, Chung KH, Oh JS, Lee CW (2013). The effect of site quality on repurchase intention in Internet shopping through mediating variables: The case of university students in South Korea. International Journal of Information Management.

[36] Bagozzi RP, Yi Y (1988). On the evaluation of structural equation models. JAMS.

[37] Fornell C, Larcker DF (1981). Structural Equation Models with Unobservable Variables and Measurement Error: Algebra and Statistics. Journal of Marketing Research.

[38] Hair JF, Black WC, Babin BJ, Anderson RE (2010). Multivariate Data Analysis, 7th edition.

[39] Podsakoff PM, Organ DW (1986). Self-Reports in Organizational Research: Problems and Prospects. Journal of Management.

[40] Preacher KJ, Hayes AF (2008). Asymptotic and resampling strategies for assessing and comparing indirect effects in multiple mediator models. Behav Res Methods.

[41] MacKinnon DP, Lockwood CM, Hoffman JM, West SG, Sheets V (2002). A comparison of methods to test mediation and other intervening variable effects. Psychol Methods.

[42] Audrain-Pontevia A, Menvielle L (2018). Do online health communities enhance patient-physician relationship? An assessment of the impact of social support and patient empowerment. Health Serv Manage Res.

[43] Beldad AD, Hegner SM (2017). Expanding the Technology Acceptance Model with the Inclusion of Trust, Social Influence, and Health Valuation to Determine the Predictors of German Users’ Willingness to Continue using a Fitness App: A Structural Equation Modeling Approach. International Journal of Human–Computer Interaction.

[44] Wu B (2018). Patient Continued Use of Online Health Care Communities: Web Mining of Patient-Doctor Communication. J Med Internet Res.

[45] Anderson RM, Funnell MM, Fitzgerald JT, Marrero DG (2000). The Diabetes Empowerment Scale: a measure of psychosocial self-efficacy. Diabetes Care.

[46] Heravi A, Mubarak S, Raymond Choo K (2018). Information privacy in online social networks: Uses and gratification perspective. Computers in Human Behavior.

