# Supplementary material for: Influence Mechanism of the Affordances of Chronic Disease Management Apps on Continuance Intention: Questionnaire Study
Source: JMIR Mhealth Uhealth. 2021 May 13;9(5):e21831. doi: 10.2196/21831 (PMC8160810; doi:10.2196/21831)
Supplement: Multimedia Appendix 1 [file mhealth_v9i5e21831_app1.docx]

**Appendix 1:** The detailed information of the hypotheses.

### Perceived affordances and gratifications

According to Scheepers and Middleton’s [1] research on the use of IT products, the different affordances of IT products are conducive to satisfying the corresponding needs of users; that is, utilitarian, connection, and hedonic affordances satisfy utilitarian, connective, and hedonistic needs. Combining previous research on UGT with the original intention of CDM apps, this study assumes the gratification of utilitarian needs is mainly reflected in quick, convenient, and efficient access to health information and health management. The gratification of connection-related needs is mainly reflected in information and social interaction. Lastly, the gratification of hedonic needs is mainly reflected in the enjoyment function. Thus, we propose the following hypotheses:

H1a: Perceived connection affordances positively affect users’ social interactivity gratification.

H1b: Perceived connection affordances positively affect users’ informativeness gratification.

H2a: Perceived utilitarian affordances positively affect users’ informativeness gratification.

H2b: Perceived utilitarian affordances positively affect users’ technology gratification.

H2c: Perceived utilitarian affordances positively affect users’ function gratification.

H3a: Perceived hedonic affordances positively affect users’ function gratification.

H3b: Perceived hedonic affordances positively affect users’ enjoyment gratification.

### Gratifications and health empowerment

CDM apps can meet users’ needs for social interaction through social functions, and users can obtain social and emotional support from doctors or peers through such channels [2]. The social support obtained by users can effectively relieve inner pressure, help them cope with emotional problems, and improve communication to achieve health empowerment [3]. Therefore, meeting users’ needs for social interaction can help promote their awareness of health empowerment. In addition, meeting users’ informativeness needs can help improve their knowledge, health literacy, and self-management ability; health empowerment is based on such cultivation of users’ abilities [4]. Therefore, meeting users’ demands for informativeness can support their cognition of health empowerment. Thus, we propose the following hypotheses:

H4a: Users’ social interactivity gratification positively affects their cognition of health empowerment.

H4b: Users’ informativeness gratification positively affects their cognition of health empowerment.

Technology gratification involves whether a CDM app can help users achieve effective health management conveniently, easily, and inexpensively, anywhere, anytime. It is mainly reflected in the convenience, ease of use, and ubiquity of the app. Ubiquity helps users manage their health at any time, free of the constraints of time and place imposed by traditional offline management, giving them better control over their health. It satisfies autonomy needs and is conducive to self-determination [5], which health empowerment emphasizes. Therefore, we propose the following hypothesis:

H4c: Users’ technology gratification positively affects their cognition of health empowerment.

Self-management emphasizes the satisfaction of the user’s autonomy needs and control over his or her own health. According to self-determination theory, this is beneficial to the user’s self-determination [5], which health empowerment emphasizes. Therefore, the satisfaction of users’ self-health management needs is conducive to their cognition of health empowerment. Thus, we propose the following hypothesis:

H4d: Users’ function gratification (self-management) positively affects their cognition of health empowerment.

Enjoyment gratification involves the degree to which the CDM app can reduce users’ anxiety and help them relax so they can more easily recover [6, 7]. Meeting demands of this kind is conducive to relieving patients’ pressures, maintaining positive and healthy emotions, improving confidence in curing diseases (i.e., promoting self-efficacy), and promoting patience. Anderson and Funnell, et al. [3] suggested that health empowerment can be achieved by managing stress, coping with emotions, and improving patients’ self-efficacy (which is closely related to health empowerment). Thus, we propose the following hypothesis:

H4e: Users’ enjoyment gratification has a positive influence on health empowerment.

### Gratifications and continuance intention

The gratification of user needs is a key antecedent of information systems users’ continuance intention. Prior research has used UGT to explore the factors influencing continuance intention and has found that gratification often positively predicts continuance intention. For example, in the context of WeChat, Gan and Li [6] found that users’ gratification from technology and information sharing functions positively affected their continuance intention. Furthermore, meeting users’ different needs can help establish trust between the platform and its users, thus promoting continuance intention [8, 9]. In addition, satisfying users’ different needs is conducive to enhancing their emotional attachment to the platform and sense of authentic experience, thus increasing their willingness to continue using it [7]. Therefore, we propose the following hypotheses:

H5a: Users’ social interactivity gratification positively affects continuance intention.

H5b: Users’ informativeness gratification positively affects continuance intention.

H5c: Users’ technology gratification positively affects continuance intention.

H5d: Users’ function gratification positively affects continuance intention.

H5e: Users’ enjoyment gratification positively affects continuance intention.

### Health empowerment and continuance intention

Health empowerment is an effective strategy for promoting individual health, and self-management is an important aspect of Health empowerment. Cheng, et al. [10] used randomized controlled trials to study the effects of Health empowerment on Chinese diabetes patients. The results showed that health-empowered groups could achieve better self-management. Therefore, when users have a higher level of empowerment, they are willing to self-manage [11]. CDM apps provide a channel for users to effectively manage themselves. When users have a higher awareness of empowerment, they are more willing to manage themselves, and intention of usage is stronger [11]. Moreover, self-determination theory holds that individuals tend to develop oriented toward an environment conducive to self-determination [12], and HE offers individuals an environment of self-determination. Thus, individuals are more inclined toward situations that empower them. Accordingly, we propose the following:

H6: Users’ cognition of health empowerment positively affects their continuance intention.

[1] Scheepers, R. and C. Middleton, Personal ICT Ensembles and Ubiquitous Information Systems Environments: Key Issues and Research Implications*.* Communications of the Association for Information Systems, 2013. 33: 381-392.

[2] Wu, T., et al., The Effect of Doctor-Consumer Interaction on Social Media on Consumers' Health Behaviors: Cross-Sectional Study*.* Journal of Medical Internet Research, 2018. 20(2): e73. PMID: 29490892

[3] Anderson, R.M., et al., The Diabetes Empowerment Scale: a measure of psychosocial self-efficacy*.* Diabetes Care, 2000. 23(6): 739-43. PMID: 10840988

[4] Anderson, R.M., et al., Patient empowerment. Results of a randomized controlled trial*.* Diabetes Care, 1995. 18(7): 943-9. PMID: 7555554

[5] Deci, E.L. and R.M. Ryan, Self-Determination Theory: A Macrotheory of Human Motivation, Development, and Health*.* Canadian Psychology-Psychologie Canadienne, 2008. 49(3): 182-185.

[6] Gan, C.M. and H.X. Li, Understanding the effects of gratifications on the continuance intention to use WeChat in China: A perspective on uses and gratifications*.* Computers in Human Behavior, 2018. 78: 306-315.

[7] Kim, M.J., C.-K. Lee, and N.S. Contractor, Seniors' usage of mobile social network sites: Applying theories of innovation diffusion and uses and gratifications*.* Computers in Human Behavior, 2019. 90: 60-73.

[8] Aladwani, A.M., Compatible quality of social media content: Conceptualization, measurement, and affordances*.* International Journal of Information Management, 2017. 37(6): 576-582.

[9] Nevzat, R., et al., Role of social media community in strengthening trust and loyalty for a university*.* Computers in Human Behavior, 2016. 65: 550-559.

[10] Cheng, L., et al., Efficacy of the Diabetes Empowerment Self-management Interactive Research (DESIRE) programme in Chinese patients with poorly controlled type 2 diabetes: a randomised controlled trial*.* Lancet, 2016. 388: 9-9.

[11] Schunk, D.H. and B.J. Zimmerman, Social origins of self-regulatory competence*.* Educational Psychologist, 1997. 32(4): 195-208.

[12] Deci, E.L. and R.M. Ryan, *Causality Orientations Theory*, in *Intrinsic Motivation and Self-Determination in Human Behavior*. 1985, Springer US: Boston, MA. p. 149-175.
