# Supplementary material for: Influence Mechanism of the Affordances of Chronic Disease Management Apps on Continuance Intention: Questionnaire Study
Source: JMIR Mhealth Uhealth. 2021 May 13;9(5):e21831. doi: 10.2196/21831 (PMC8160810; doi:10.2196/21831)
Supplement: Multimedia Appendix 2 [file mhealth_v9i5e21831_app2.docx]

**Appendix 2.** Measurement instruments.

**Perceived connective affordances**

1. The chronic disease management App has some features that support me to communicate with others.

2. The chronic disease management App has some features that support me to share my experience with others

3. The chronic disease management App has some features that support me to seek other people's opinions

**Perceived utilitarian affordances**

1. The chronic disease management App has some features to support me to develop a health management plan.

2. The chronic disease management App has some features that support me to learn about health management.

3. The chronic disease management App has some features that support me to collect information related to health management.

4. The chronic disease management App has some features that support me to test my health status.

**Perceived hedonic affordances**

1. The chronic disease management App has some features that support my relaxation spirit.

2. The chronic disease management App has some features that allow me to kill time in my spare time.

3. The chronic disease management App has some features that can help me get rid of anxiety and become happy.

**Social interactivity gratification**

1. I am satisfied with the chronic disease management App in terms of allowing me to build relationships with other users.

2. The chronic disease management App is satisfying me in helping me maintain social relationships with other users.

3. The chronic disease management App is satisfying me in helping me make new friends.

4. The chronic disease management App is satisfying me in promoting my social relationship with other users.

**Informativeness gratification**

1. The diversity of the media information format of the chronic disease management App is satisfactory to me.

2. The diversity of the electronic communication channels of the chronic disease management App is satisfactory to me.

3. I am satisfied to use the chronic disease management App to share various health knowledge.

4. I am satisfied to use of chronic disease management App to search for various health information.

**Technology gratification**

1. I like the chronic disease health management app because I can manage my health at any time.

2. Chronic disease management App is the simplest and most cost-effective way to manage health

3. It's easy for me to use the chronic disease management App for a long time.

**Functions gratification**

1. The chronic disease management App is satisfying me in guiding me to manage my health.

2. The chronic disease management App is satisfying me in helping me customize the treatment plan.

3. The chronic disease management App is satisfying me in helping me learn health management knowledge.

**Enjoyment gratification**

1. I am happy by using the Chronic Disease Management App to conduct health management related activities.

2. I am in a good mood by using the Chronic Disease Management App to conduct health management related activities.

3. It is interesting for me to use the chronic disease management App to carry out activities related to health management.

4. I can maintain a happy mood by using the Chronic Disease Management App to conduct health management related activities.

**Health empowerment (meaning, competence, self-determination and impact)**

**Meaning**

1. It is very important for me to use the chronic disease management app to manage my health.

2. Using the chronic disease management App to meet my health needs is of unique significance to me.

3. It is meaningful for me to use the chronic disease management app to manage my own health.

**Competence**

1. I am confident in my ability to meet my health needs by using the chronic disease management app.

2. I have mastered some of the necessary skills to meet my health needs by using the chronic disease management app.

3. I am sure I have the ability to meet my health needs by using the chronic disease management app.

**Self-determination**

1. I have great autonomy in how to manage health in the process of using chronic disease management App.

2. I can decide how to carry out health management in the process of using the chronic disease management App.

3. I have a considerable opportunity to decide independently how to conduct health management in the process of using the chronic disease management App.

**Impact**

1. My impact on how to manage my own health is great in the process of using the chronic disease management App.

2. I have great control over how to manage my health in the process of using the chronic disease management app.

3. I have significant influence in deciding how to manage my own health in the process of using the chronic disease management App.

**Continuance intention**

1. I plan to continue using the chronic disease management app in the future.

2. I will try to use the chronic disease management app in my daily life.

3. I will continue to use the chronic disease management app as always.
