# Supplementary material for: Influence Mechanism of the Affordances of Chronic Disease Management Apps on Continuance Intention: Questionnaire Study
Source: JMIR Mhealth Uhealth. 2021 May 13;9(5):e21831. doi: 10.2196/21831 (PMC8160810; doi:10.2196/21831)
Supplement: Multimedia Appendix 3 [file mhealth_v9i5e21831_app3.docx]

**Appendix 3:** The results of convergence validity and reliability.

| Construct | Item | VIF | Loading | T | Cronbach’s α | Composite reliability | Average variance extracted |
| --- | --- | --- | --- | --- | --- | --- | --- |
| Perceived connection affordances (PCA) | PCA1 | 1.664 | 0.859 | 52.353 | 0.763 | 0.862 | 0.676 |
|  | PCA2 | 1.478 | 0.827 | 36.369 |  |  |  |
|  | PCA3 | 1.544 | 0.779 | 20.970 |  |  |  |
| Perceived utilitarian affordances (PUA) | PUA1 | 1.435 | 0.773 | 30.161 | 0.722 | 0.827 | 0.544 |
|  | PUA2 | 1.310 | 0.745 | 22.525 |  |  |  |
|  | PUA3 | 1.354 | 0.721 | 20.271 |  |  |  |
|  | PUA4 | 1.369 | 0.711 | 18.484 |  |  |  |
| Perceived hedonic affordances (PHA) | PHA1 | 1.682 | 0.864 | 58.636 | 0.740 | 0.852 | 0.657 |
|  | PHA2 | 1.404 | 0.75 | 23.712 |  |  |  |
|  | PHA3 | 1.457 | 0.814 | 38.106 |  |  |  |
| Social interactivity gratification (SIG) | SIG1 | 1.426 | 0.764 | 24.642 | 0.740 | 0.836 | 0.561 |
|  | SIG2 | 1.474 | 0.791 | 27.564 |  |  |  |
|  | SIG3 | 1.361 | 0.738 | 21.357 |  |  |  |
|  | SIG4 | 1.386 | 0.700 | 15.458 |  |  |  |
| Informativeness gratification (IG) | IG1 | 1.459 | 0.725 | 20.770 | 0.799 | 0.869 | 0.624 |
|  | IG2 | 1.609 | 0.788 | 26.831 |  |  |  |
|  | IG3 | 1.793 | 0.837 | 44.662 |  |  |  |
|  | IG4 | 1.646 | 0.806 | 35.213 |  |  |  |
| Technology gratification (TG) | TG1 | 1.592 | 0.840 | 42.511 | 0.715 | 0.840 | 0.638 |
|  | TG2 | 1.363 | 0.789 | 27.155 |  |  |  |
|  | TG3 | 1.370 | 0.765 | 24.798 |  |  |  |
| Function gratification (FG) | FG1 | 1.559 | 0.834 | 47.524 | 0.775 | 0.870 | 0.690 |
|  | FG2 | 1.611 | 0.814 | 35.536 |  |  |  |
|  | FG3 | 1.620 | 0.843 | 51.004 |  |  |  |
| Enjoyment gratification (EG) | EG1 | 1.597 | 0.768 | 25.551 | 0.774 | 0.855 | 0.596 |
|  | EG2 | 1.421 | 0.78 | 30.306 |  |  |  |
|  | EG3 | 1.614 | 0.782 | 28.030 |  |  |  |
|  | EG4 | 1.445 | 0.753 | 24.171 |  |  |  |
| Health empowerment  (HE) | C1 | 1.414 | 0.801 | 27.660 | 0.757 | 0.861 | 0.673 |
|  | C2 | 1.388 | 0.799 | 29.985 |  |  |  |
|  | C3 | 1.370 | 0.789 | 30.202 |  |  |  |
|  | I1 | 1.479 | 0.804 | 26.021 |  |  |  |
|  | I2 | 1.445 | 0.771 | 22.206 |  |  |  |
|  | I3 | 1.415 | 0.839 | 31.989 |  |  |  |
|  | M1 | 1.494 | 0.815 | 36.382 |  |  |  |
|  | M2 | 1.571 | 0.808 | 30.489 |  |  |  |
|  | M3 | 1.466 | 0.827 | 41.195 |  |  |  |
| Continuance intention  (CI) | CI1 | 1.527 | 0.820 | 33.275 | 0.719 | 0.842 | 0.640 |
|  | CI2 | 1.333 | 0.777 | 27.323 |  |  |  |
|  | CI3 | 1.429 | 0.803 | 31.050 |  |  |  |
