# Supplementary material for: Influence Mechanism of the Affordances of Chronic Disease Management Apps on Continuance Intention: Questionnaire Study
Source: JMIR Mhealth Uhealth. 2021 May 13;9(5):e21831. doi: 10.2196/21831 (PMC8160810; doi:10.2196/21831)
Supplement: Multimedia Appendix 4 [file mhealth_v9i5e21831_app4.docx]

**Appendix 4:** Discriminant validity (The values on the diagonal are the square root of AVE).

|  | PCA | PUA | PHA | SIG | IG | TG | FG | EG | HE | CI |
| --- | --- | --- | --- | --- | --- | --- | --- | --- | --- | --- |
| PCA^a^ | 0.822 |  |  |  |  |  |  |  |  |  |
| PUA^b^ | 0.337 | 0.738 |  |  |  |  |  |  |  |  |
| PHA^c^ | 0.367 | 0.199 | 0.811 |  |  |  |  |  |  |  |
| SIG^d^ | 0.335 | 0.223 | 0.336 | 0.749 |  |  |  |  |  |  |
| IG^e^ | 0.4 | 0.489 | 0.342 | 0.343 | 0.79 |  |  |  |  |  |
| TG^f^ | 0.24 | 0.425 | 0.301 | 0.248 | 0.36 | 0.799 |  |  |  |  |
| FG^g^ | 0.374 | 0.512 | 0.354 | 0.332 | 0.5 | 0.453 | 0.83 |  |  |  |
| EG^h^ | 0.387 | 0.348 | 0.573 | 0.341 | 0.47 | 0.337 | 0.392 | 0.772 |  |  |
| HE^i^ | 0.444 | 0.567 | 0.463 | 0.409 | 0.563 | 0.608 | 0.545 | 0.554 | 0.82 |  |
| CI^j^ | 0.231 | 0.516 | 0.293 | 0.386 | 0.449 | 0.44 | 0.459 | 0.298 | 0.561 | 0.8 |

^a^PCA**:** Perceived connection affordances ^f^TG**:** Technology gratification

^b^PUA: Perceived utilitarian affordances ^g^FG: Function gratification

^c^PHA: Perceived hedonic affordances ^h^EG: Enjoyment gratification

^d^SIG: Social interactivity gratification ^i^HE: Health empowerment
